# The association between influenza vaccination and socioeconomic status in high income countries varies by the measure used: a systematic review

**DOI:** 10.1186/s12874-019-0801-1

**Published:** 2019-07-17

**Authors:** Kelsey Lucyk, Kimberley A. Simmonds, Diane L. Lorenzetti, Steven J. Drews, Lawrence W. Svenson, Margaret L. Russell

**Affiliations:** 1 Department of Community Health Sciences, Cumming School of Medicine, University of Calgary, 3280 Hospital Dr NW, Calgary, AB T2N 3Z6 Canada; 2 Alberta Ministry of Health, 10025 Jasper Avenue, Edmonton, AB T5J 1S6 Canada; 3 Health Sciences Library, University of Calgary, 3330 Hospital Drive NW, Calgary, AB T2N 4N1 Canada; 4 Medical Microbiology, Canadian Blood Services, 1800 Alta Vista Dr, Ottawa, ON K1G 4J5 Canada; 5 Departments of Laboratory Medicine & Pathology, University of Alberta, 8440 - 112 St, Edmonton, AB T6G 2J2 Canada; 6 Division of Preventive Medicine & School of Public Health, University of Alberta, Edmonton, 5-22F, University Terrace, 8303 112 ST NW, Edmonton, AB T6G 1K4 Canada

**Keywords:** Influenza, Vaccination, Socioeconomic status

## Abstract

**Background:**

The purpose of this paper is to systematically review the literature on the relationship between socioeconomic status (SES) and influenza immunization and to examine how certain measures of SES may influence interpretations of this relationship.

**Methods:**

We conducted a systematic review of existing peer-reviewed literature to evaluate the above relationship in the general population. Electronic databases (MEDLINE and EMBASE) were searched from January 2012 to May 2017 to identify English-language studies relevant to this review. Studies were included where influenza vaccination was explicitly reported as the dependent variable and SES as the independent variable. We limited our review to measures of SES that focus on education, income, social class, occupation, and deprivation. Studies that measured SES using other variables (e.g., race, ethnicity, geographic location, rural or urban status, or insurance status) were excluded. Studies were also excluded if they did not report on the human population or did not analyze original data. The population of interest included all age groups, levels of health status, and sociodemographic backgrounds. The review was also limited to World Bank high-income countries. Two authors independently screened full-text articles after obtaining a Kappa score of *K =* 0.867. The methodological quality of manuscripts was assessed using the appraisal tools developed by the Joanna Briggs Institute. Results were qualitatively reported and synthesized.

**Results:**

Of the 42 articles included in this review, 52.4% (*n* = 22) found that higher levels of SES resulted in higher levels of influenza vaccination; 4.5% (*n* = 2) reported a negative association; and 14.3% (*n* = 6) found no association. Just over a quarter (26.2%, *n* = 12) of articles reported mixed results.

**Conclusions:**

There was consistently a relationship between SES and influenza immunization, which varied according to how SES was measured. It is recommended that authors be explicit in defining the SES concept they are trying to capture and that they utilize multiple measures of SES (e.g., education, income, class).

**Electronic supplementary material:**

The online version of this article (10.1186/s12874-019-0801-1) contains supplementary material, which is available to authorized users.

## Background

Worldwide, public health authorities have implemented vaccination programs targeting groups at high-risk of morbidity and mortality for influenza. The World Health Organization (WHO) recommends annual vaccination for pregnant women, children between the ages of 6 months to 5 years, the elderly over the age of 65 years, individuals with chronic medical conditions, and health-care workers [1]. In some jurisdictions, influenza vaccination is provided at low-cost or no-cost (out of pocket) to certain individuals. Canada offers universal coverage for medically necessary health care services (i.e., “universal publicly funded healthcare”) [2]. As of 2018, 10 of Canada’s 13 provinces and territories provide the vaccine free of charge to all residents; the remainder offer free vaccine to targeted high-risk groups [3].

The relationship between socioeconomic status (SES) and health is well-documented worldwide for a number of outcomes [4], including those related to influenza. Disparities in influenza-related hospitalizations and deaths may occur in certain populations where social determinants of health influence an individual’s exposure to a disease, their risk behaviours, or their options for treatment and prevention [5]. Surveillance data in the United States (US) have shown higher rates of influenza hospitalizations in areas with high levels of poverty, population density, crowded housing conditions, and female-headed households [5, 6].

As Nagata et al. (2013) found in a systematic review on the association between social determinants of health and seasonal influenza vaccination in the elderly population, the direction of the association between vaccination and SES varies [7]. The authors found that elderly persons from higher SES backgrounds sometimes had higher rates of vaccinations (e.g., in areas where policies and programs had been implemented to increase vaccination among vulnerable and high-risk groups), but not always [7]. Given the mixed findings reported by the above review, there is a need to revisit the literature regarding the relationship between SES and influenza vaccination with respect to the general population, including children, adults, and the elderly.

There is a lack of consensus in the literature on how SES should be measured [8]. SES is a complex social, economic, and political concept that cannot be measured directly. Instead, SES is measured indirectly by using the proxy measures of class (e.g., economic resources), prestige (e.g., community ranking), occupation (e.g., occupational class), and/or education (e.g., education level) [9, 10]. We anticipate that the different ways of measuring SES may mask or intensify associations between SES and vaccination status and that associations may differ depending on whether the vaccine is universally available and publicly funded.

The purpose of this review was first to systematically review and synthesize the literature to answer the following research question: Is there a relationship between SES and influenza immunization status? Second, we wished to determine to what extent specific measures used to capture SES influenced the relationship between SES and influenza immunization status.

## Methods

### Protocol and eligibility criteria

We searched the literature to identify quantitative studies where the outcome measure was influenza vaccination and the independent variable was SES. The PRISMA guidelines for reporting systematic reviews have been followed throughout this manuscript [11]. The study protocol for this review has not been previously published or registered [11].

### Information sources

We searched OVID Embase and Ovid MEDLINE databases from January 2012 to May 2017 to identify relevant studies. We consulted a medical librarian to develop the search strategy.

### Search

Searches were developed that combined terms from two themes: SES (occupation, income, education, employment, class, or social and material deprivation) [8] and influenza immunization (influenza, immunization, immunisation, vaccine, vaccination). Terms were searched as both title/abstract words and database-specific subject headings (Additional file 1). The search included only empirical English-language articles published from January 2012 to May 2017. No study design filters were applied.

### Study selection

**Fig. 1:**
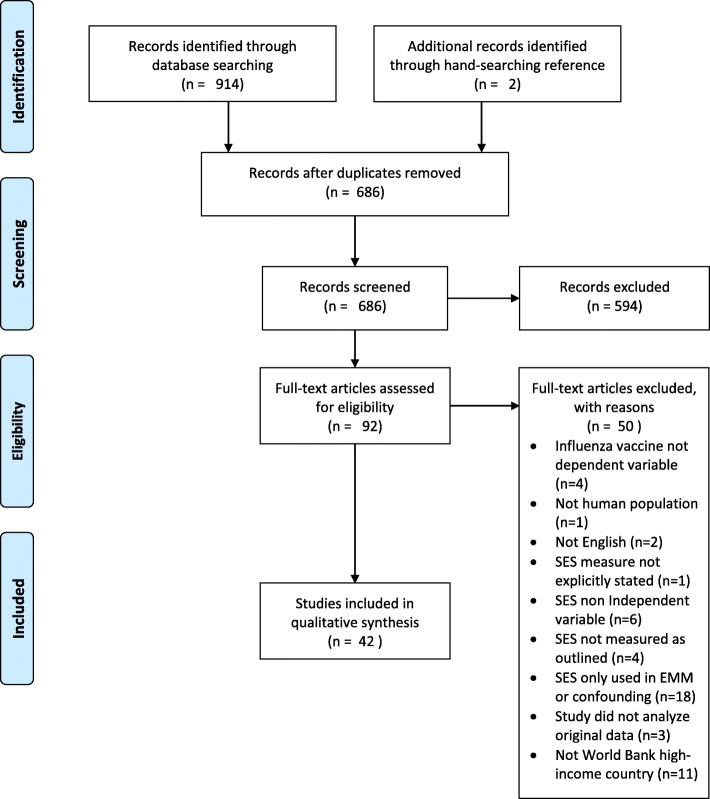
PRISMA flow diagram of study selection

Two authors (KL, MLR) independently screened abstracts in duplicate for eligibility. Using a sample of eligible articles, these authors developed a full-text review sheet to reach agreement on article inclusion from a sample of 15 articles (*K* = 0.867). Following this, one author (KL) independently screened full-text articles for inclusion. Studies were not excluded on the basis of summary measures used (e.g., odds ratios, means), provided that they quantitatively reported vaccination rates by SES level. Studies were included where: 1) the primary outcome was influenza vaccination, measured in any way (e.g., self-report, administrative records, health records) as a dependent variable; 2) SES was an independent variable, explicitly defined, and expressed as social class, socioeconomic status, socioeconomic position, occupational class, educational attainment, income, poverty, deprivation index, neighbourhood-based measures (e.g., postal code to show deprivation areas), and/or employment status (Fig. 1); and 3) studies reported on data from World Bank high-income countries [12]. Studies were excluded if they: 1) did not report influenza vaccination as the primary outcome or independent variable; 2) did not report on the human population; 3) were not published in the English language; 4) did not explicitly state the SES measure used; 5) did not measure SES as outlined above; 6) used SES to assess only for effect measure modification or confounding; 7) did not analyze original data; 8) did not have a comparison group, if an analytic study; or, 9) did not concern a World Bank high-income country. The reference lists of included articles were hand-searched to identify relevant articles that met the search and inclusion criteria.

### Data extraction

Two authors (KL, MLR) developed a data extraction form, which one author (KL) used to extract data from the included studies. Data included: the population (e.g., age group, health status, sex), intervention (e.g., SES concept used, measure of SES, level of SES measure used, analysis, strength of association), comparison group (e.g., unvaccinated adults), study details (e.g., study purpose, study design, data source used), setting (e.g., country, year data collected, city), and results (e.g., findings on the association between influenza vaccination and SES). For each association reported, we recorded the variables that study authors adjusted for in their analysis, such as age and sex. Many studies adjusted for numerous “sociodemographic variables” in their multivariable analyses, which, as described in the legend of Tables 1, 2 and 3, included age, sex, place of residence, education level, and rural residence. Tables 1, 2 and 3 also report details on study findings, summary measures, and the level of significance for each association found.

**Table 1:** Association between influenza vaccination and SES, by single-measure SES

Study; Country	SES Concept (Level of SES measure)	Description SES measure	Adjusted mediating factors^a^	Data source; Data type (Year collected)	Results
Education (*n* = 6; 3 positive associations, 1 negative association, 2 no association)					
Barbadoro 2013 [13]; Italy	Education (Individual)	Level of education level (low, medium, high, 3-yr university degree, more than 4 years university degree, post-graduate)	Sociodemographic and clinical variables	Health and Use of Health Care in Italy; National Survey (2004–05)	Positive association. Healthcare workers in Italy with low (0.57OR, 0.39–0.81CI), medium (0.51OR, 0.37–0.71CI), or high (0.54OR, 0.39–0.71CI) education levels were less likely to be vaccinated compared to those with a post-graduate degree.
Cohen 2012 [14]; US	Education (Individual)	Low (less than HS, HS, or GED) or high (some college or college diploma) levels of education	Sociodemographic, health, clinical, and health belief variables	Participants of a RCT on flu transmission; Study in Washington Heights, New York (2006)	Positive association. Adults with higher levels of education were more likely to be vaccinated (1.20OR, 1,05–1,37CI) than those with lower levels. No significant relationship was found for children.
Henninger 2015 [15]; US	Education (Individual)	Education level (HS or GED or less, some college, bachelor’s degree, master’s degree or higher)	Sociodemographic variables, obstetric characteristics, survey items, and vaccination status	Pregnancy and Influenza Project; Large cohort of pregnant women seeking care from Kaiser Pemanente Northwest and Northern California (2010–11)	No association. Education level was not significantly associated with vaccination during pregnancy after adjusting for race and provider recommendation.
Jimenez-Trujillo 2015 [16]; Spain	Education (Individual)	Education level (primary, secondary, university)	Sociodemographic variables, health status and clinical variables	European Health Survey for Spain and Spanish National Health Survey; National Survey (2009; 2011)	No association. There was no significant association found between education level and influenza vaccine among persons aged 40–69 years with diabetes.
Lorenz 2013 [17]; US	Education (Individual)	Education level (less than HS, HS, more than HS)	Not reported	Patient survey and electronic medical records; Statewide survey and electronic medical records (2010–12)	Negative association. Mentally ill patients in Alabama with more than a high school education were less likely (0.29OR, 0.09–0.96CI) to be vaccinated compared to those with less than a high school education.
Simon 2016 [18]; US	Education (Individual)	Parents’ highest level of education (less than HS, HS graduate or GED, some college or associated degree, technical school degree, college degree or more)	Not reported	National Health Interview Survey; National survey (2005–13)	Positive association. Children with asthma whose parents had a college degree or higher (reference group) were more likely to receive influenza vaccination compared to those with parents who had some college, an associate’s degree, or some technical school (0.62 OR, 0.42–0.91 CI).
Occupational class (*n* = 1; 1 positive association)					
Cleary 2014 [19]; Ireland	Class (Individual)	Socioeconomic group (occupational class): home duties, professional / manager / employer / non-manual, manual, unemployed, non-classifiable	Sociodemographic and pregnancy-related clinical variables; no adjustment was done in calculation for vaccination by SES group	Antenatal booking interview hospital data; Medical and clinical admission records (2009–10)	Positive association. Pregnant women from the professional / manager / employer group were more likely to be vaccinated against H1N1 than women in all other SES groups: home duties (0.61OR, 0.52–0.70CI), non-manual (0.81OR, 0.72–9.92CI), manual (0.54OR, 0.42–0.69CI), unemployed (0.77OR, 0.64–0.93CI), or non-classifiable (0.69OR, 0.55–0.87CI).
Income or poverty (*n* = 5; 5 positive associations)					
Campitelli 2012 [20]; Canada	Income (Neighbourhood)	Linked postal code of residence to mean household income quintile in area (Q1 = lowest SES, Q5 = highest SES)	Sociodemographic and clinical variables	Hospital discharge, abstracts, physician claims, and other databases; Linked administrative data (2002–12)	Positive association. Infants from higher SES quintiles were more likely to be fully vaccinated against influenza during their first eligible winter, compared to children from lower SES areas (Q2 = 1.08OR, 1.04–1.11CI, Q3 = 1.12OR, 1.09–1.16CI, Q4 = 1.20OR, 1.23–1.32CI). The association remained significant for SES quintiles 3, 4, and 5 among low-birth weight infants.
Fox 2014 [21]; US	Income (Individual)	Federal poverty level for 2012: income above 200% the poverty level, income equal to or less than 200% poverty level	None	National Health Interview Survey; National survey (2011–12)	Positive association. Adults with a family income above 200% the federal poverty level were significantly more likely to receive an influenza vaccination compared to those below it (1.3PR, 1.3–1.3CI)
Lau 2013 [22]; US	Income (Household)	Household income percent of federal poverty level: 0–99%, 100–199%, 200–299, 300% or more	Sociodemographic variables, insurance status, and usual source of care	California Health Interview Survey; State survey (2005; 2007)	Positive association. After adjusting for gender, race/ethnicity, income, insurance status, and usual source of care, young adults aged 18 to 26 years with household incomes 200–299% above the federal poverty limit (1.57OR, 1.11–2.21CI) were more likely to be vaccinated compared to young adults of the same age with household incomes 0–99% FPL.
Narciso 2012 [23]; US	Income (Neighbourhood)	Neighbourhood poverty level (high [> 30% residents living below poverty], medium [20–29.9% residents living below poverty], low [less than 20% living below poverty level])	Not reported	Vaccination data from school-located vaccination campaign; New York City schools (2009–10)	Positive association. The authors report lower levels of vaccination in boroughs with higher levels of poverty and provide the following vaccination prevalence rates: Manhattan (27.5%), Bronx (18.4%), Brooklyn (19.4%), Staten Island (16.9%), Queens (24.2%).
Villarroel 2016 [24]; US	Income (Household)	Income level (poor: below FPL, near-poor: 100% to less than 200% FPL, not poor: 200% or greater FPL)	Not reported	National Health Interview Survey; National survey (2015)	Positive association. Among adults diagnosed with diabetes, 50.9% who were poor had received an influenza vaccine, compared to those who were 57.8% near-poor and 65.9% not-poor. This linear trend was significant at *p* < 0.05.

*Acronyms*: *CI* confidence interval, *GED* General Equivalency Diploma, *FPL* Federal Poverty Level, *OR* odds ratio, *HS* highschool, *PR* prevalence ratio, *SES* socioeconomic status, *US* United States

^a^Sociodemographic variables included: age, sex, place of residence, education level, rural residence. Clinical variables included: underlying chronic disease, preventive health practices, health status, health care worker, primary care provider, continuity of care, low birth weight, respiratory illness. Health belief variables included: knowledge, attitudes, practice

**Table 2:** Association between influenza vaccination and SES, by two-measure SES

Study; Country	SES Concept (Level of SES measure)	Description SES measure	Adjusted mediating factors^a^	Data source; Data type (Year collected)	Results
Education and social or occupational class (*n* = 2; 1 positive association, 1 no association)					
LaVela 2012 [25]; US	Education and employment (Individual)	Education level: did not graduate HS, HS or more Employment status: currently employed for wages, or not	Sociodemographic characteristics, characteristics of multiple sclerosis, comorbidities	Behavioral Risk Factor Surveillance System and Multiple Sclerosis Health Care Questionnaire; National survey (2003)	No association. There was no significant association between influenza vaccination, being in paid employment, or having completed high school among men age 50 or older with multiple sclerosis.
Lu 2015 [26]; US	Education and employment (Individual)	Employment status (employed, unemployed, not in work force) Education level (less than HS, HS, college or more)	Sociodemographic variables and health service variables	National Health Information Survey; National survey (2012)	Positive association. Adults aged 19–64 with college education or higher were more likely (1.18, 1.09–1.27CI) to receive a vaccination compared to persons with HS, as were persons aged 65 and older (1.11APR, 1.04–1.18CI). Unemployed adults were less likely to be vaccinated compared to employed adults (0.89APR, 0.80–1.00CI), but among the elderly, unemployed persons were more likely to be vaccinated (1.10APR, 1.03–1.17CI).
Education and income or poverty (*n* = 15; 7 positive associations; 6 mixed findings; 2 no association)					
Barbadoro 2016 [27]; Italy	Education and income (Individual)	Education level (low, medium-low, medium-high, high) Wealth (1 = low, 2, 3, 4 = high)	Sociodemographic and clinical variables	Health and Health Care use in Italy; National survey (2004–05; 2013)	Mixed findings. Obese persons with medium-high levels of education were less likely to be vaccinated compared to persons with low levels of education in two age groups: 18–64 years (0.77OR, 0.62–0.96CI) and 64 and older (0.79OR, 0.63–0.98CI). No association was found with income.
Blackwell 2015 [28]; US	Education and income (Individual and household)	Highest level of parental education in household (less than HS, HS diploma, GED, some college, college degree) Poverty ratios for 2010: below federal threshold (less than 1.0, 1 to < 2 times threshold, 2 to < 4 times threshold, 4 times or more threshold)	Sociodemographic and health status	National Health Interview Survey; National survey (2009–10)	Positive association. Children of parents whose highest level of education was HS or GED (0.82ARR, 0.68–1.0CI) or less than a HS diploma (0.72ARR, 0.57–0.92) were less likely to be vaccinated compared to those with parents who had some post-secondary education. Children living 4 times or more above the poverty threshold were more likely to be vaccinated compared to those living less than 1 times below (0.75ARR, 0.59–0.96CI), 1 to < 2 times below (0.69ARR, 0.57–0.84CI), 2 to < 4 times below (0.77ARR, 0.66–0.90CI). There was no association between education and income and receipt of a second pH1N1 vaccination.
CDC 2013 [29]; US	Education and income (Individual and household)	Level of education (less than 12 years, 12 years, more than 12 years) Federal poverty level for 2010 (at or below the poverty level, above the poverty level)	None reported	Massachusetts Pregnancy Risk Assessment Monitoring System; State survey (2009–10)	Positive association. Pregnant women with less than 12 years (56.6%, 45.0–67.5CI) of education had lower seasonal vaccination rates compared to those with 12 years (63.1%, 54.4–71.1CI) or greater than 12 years education (71.1%, 66.0–75.5CI). No association was found with pH1N1 vaccine. Pregnant women living above the federal poverty line had greater coverage of seasonal influenza vaccination (70.5%, 65.6–75.0CI) compared to those living at or below it (56.1%, 47.5–64.3%), but no association was found for pH1N1.
Dlugacz 2012 [30]; US	Education and income (Individual)	Education level (less than HS, HS, college graduate, graduate degree) Income level ($75,000 or more, $50,000–$75,000, less than $50,000)	Sociodemographic variables	Survey of postpartum women on labour and delivery service; Survey administered in 4 hospitals in Nassau County New York, and Queens County in New York City (2010)	Positive association. In the unadjusted model, each increase in level of education among postpartum women resulted in higher levels of vaccination. For income levels, the highest income group was twice as likely to have been vaccinated than the middle and low-income group. The adjusted ORs were not reported for income and education separately. When looking at the combined effects of income and education, higher levels resulted in 43–69% of pregnant women receiving a vaccine when recommended by a healthcare provider, compared to 4–10% when not recommended.
Gorska-Ciebiada 2015 [31]; Poland	Education and income (Individual)	High (above 2000 pln) or low income (2000 pln or lower) Education level (primary, secondary, technical, university)	Sociodemographic, health status, and clinical variables	Survey of elders in an internal medicine and diabetology outpatient clinic in Lodz; Survey of clinic outpatients (2012–13)	Mixed findings. Elderly outpatients with diabetes aged 65 and older were more likely to be vaccinated if they had higher income (5.34OR, 2.38–12.16CI).
Hellfritzsch 2017 [32]; Denmark	Education and income (Individual and household)	Education level, in years beyond primary school (none, less than 3 years, 3–4 years, more than 4 years, other) Annual household income in Danish Kroner (less than $99,000, $100,000–$149,000, $150,000–$249,000, $250,000–$374,999, $375,000–$524,000, more than $524,000)	Age and sex	Questionnaire from Centre for Public Health; National survey (2006)	Mixed findings. There was no association between seasonal influenza vaccination and education level among Danes ages 65–79. For income, only the middle-income group (1.10PR, 1.00–1.21CI) was associated with seasonal influenza vaccination after controlling for age and sex.
Hoeck 2013 [33]; Belgium	Education and income (Household)	Highest level of education (no degree or primary education, lower secondary, higher secondary, higher education) Household income (<€750, €750–€1000, €1000–€1500, €1500–€2500, and > €2500)	Sociodemographic variables, health status, and risk factors	Belgian Health Interview Survey; National survey (2004; 2008)	No association. There was no significant association between level of education or household income with influenza vaccination.
Kwon 2016 [34]; South Korea	Education and income (Individual and household)	Education level: low (less than 6 years) or high (more than elementary school) Household income (less than $1000 USD per month, or $1000 USD or more per month)	Sociodemographic variables, health status, and behavioural risk factor variables	Korean National Health and Nutrition Examination Survey; National survey (2007–09)	Mixed findings. Higher education was associated with higher levels of vaccination (1.27OR, 1.03–1.57CI) among the elderly 65 and older. No association with income was found.
Lee 2015 [35]; South Korea	Education and income (Individual and household)	Education level: low (elementary), middle (middle or high school), high (college or higher) Household income (low [Q1), middle [Q2, Q3], high [Q4])	Sociodemographic factors and health status variables	Korea National Health and Nutrition Examination Survey; National survey (2010–11)	Mixed findings. No significant relationship was found between influenza vaccination and education for any age group. Younger adults aged 19–50 years were less likely to be vaccinated if they were in the highest income group (0.33OR, 0.26–0.40CI), compared to the lowest. For adults age 50 or older, no significant association was found.
Lee 2012 [36]; US	Education and income (Individual)	Education level (none, 5 years or less, more than 5 years) Income ($50,000 or less, more than $50,000)	Sociodemographic variables, health status variables, education in the US and acculturation level	Survey of Korean-Americans; Nationally representative survey (2005–07)	No association. No significant associations were found between education or income and influenza vaccination among adult Korean women living in California.
Lu 2012 [37]; US	Education and income (Individual)	Education level (less than HS, HS graduate, college) Income (below $20,000, $20,000-50,000, $50,000 or higher)	Sociodemographic factors, health status variables, and insurance status	Behavioral Risk Factor Surveillance System; National survey (2009–10)	Positive association. Among healthcare personnel, adults were more likely to be vaccinated against H1N1 or seasonal influenza if they had incomes above $50,000. For non-healthcare personnel, only seasonal influenza vaccination was associated with higher income levels. For education, significant and positive associations were found for H1N1 and seasonal influenza vaccinations, whereby the highest education group had the highest proportion of vaccine.
Lu 2016 [38]; US	Education and poverty (Individual)	Education level (HS or less, some college or college graduate, above college graduate) Poverty level (at or above poverty, below poverty)	Sociodemographic variables and health status variables	National Health Information Survey; National survey (2013; 2014)	Mixed findings. Healthcare personnel with college education were significantly more likely to have received influenza vaccination compared to those with high school or less (1.27 PR, 1.11–1.46 CI). There was no relationship found with poverty and vaccination status for healthcare personnel. For non-healthcare personnel, any level of education higher than high school was significantly associated with vaccination. (College level compared to high school or less was 1.10 PR, 1.06–1,15 CI; above college 1.34 PR, 1.26–1.42 CI). Non-healthcare personnel were more likely to be vaccinated if they were at or above the poverty level (1.08 PR, 1.02–1.14 CI) compared to those below it.
Schuller 2013 [39]; US	Education and poverty (Individual)	Mother’s education level (college graduate less than 12 years, 12 years, more than 12 years, non-college graduate) Poverty status (above poverty level with $75,000 or more, or below poverty level)	Sociodemographic variables	National Immunization Survey; National survey (2008–09)	Positive association. Mothers who graduated from college were more likely to vaccinate their children compared to mothers with a high school degree (0.57OR, 0.51 to 0.63 CI). Mothers who lived above the poverty level with incomes of $75,000 or more were significantly more likely to vaccinate their children compared to those who lived above the poverty level with lower levels of income (0.64 OR, 0.58 to 0.71 CI) or below it (0.63 OR, 0.56 to 0.71 CI).
Takayama 2012 [40]; US	Education and income (Individual)	Education (less than HS, more than HS) Annual income (less than $35,000, more than $35,000)	Sociodemographic variables, health behavior variables, and physical health status	Behavioral Risk Factor Surveillance System; National survey (2009)	Positive association. Among elderly adults 60 years and older, having less than a high school education was significantly associated with lower levels of vaccination (0.95 OR, 0.92 to 0.99 CI). For older adults and adults aged 16 to 64 years, lower income was significantly associated with lower levels of vaccination.
Zhai 2017 [41]; US	Education and poverty (Individual)	Mothers education level (less than 12 years school, 12 years of school, more than 12 years of school, not a college graduate) Poverty status (above poverty level with more than $75,000 per year, above the poverty level with less than $75,000 per year, at or below the poverty level)	Not reported	National Immunization Survey Flu (NIS-Flu); National survey of households with children aged 6 months to 17 years (2015)	Positive association. In 2012–2013, higher levels of mother’s education were significantly related to vaccination among children aged 6 months to 8 years, increasingly, for every level of education. In the 2013–2014 season, only having a college degree was significantly related to vaccination, while having 12 years of school was significantly less likely to be vaccinated. Poverty status was significantly related to higher levels of vaccination for children whose parents lived above the poverty line and made more than $75,000 per year, compared to those living with lower incomes in both seasons.
Income and poverty (*n* = 1; 1 mixed findings)					
Muscoplat 2013 [42]; US	Income and poverty (Neighbourhood)	Percentage of residents in a ZIP code with incomes below the federal poverty level (<$35,000; $35,000 to <$40,000; $40,000 to $50,000; >$50,000) Percentage of residents with incomes below the poverty level (< 3.0%; 3.0 to < 5.0%; 5.0 to 8.0%; > 8.0%)	Poverty and minority status	Minnesota Immunization Information Connection; Immunization database (2009–10)	Mixed findings. There were significant differences in H1N1 vaccination rates when comparing percentage of residents living below the poverty line. The results show that generally, areas where there were greater proportions of persons living below the poverty line (5 to 8%, or 8% and higher) had higher vaccination rates than in areas where there were lower proportions (3 to < 5%, less than 3%). Median family income was significantly related to vaccination rates. In areas with higher vaccination rates (20% and above), higher income was associated with higher vaccination levels. In areas where vaccine rates were less than 20%, lower income levels were associated with higher levels of vaccination.

*Acronyms*: *APR* adjusted prevalence ratio, *ARR* adjusted risk ratio, *CDC* Centers of Disease Control and Prevention, *CI* confidence interval, *GED* General Equivalency Diploma, *OR* odds ratio, *HS* highschool, *pln* Polish Zloty, *PR* prevalence ratio, *SES* socioeconomic status, *US* United States, *USD* United States dollar

^a^Sociodemographic variables included: age, sex, place of residence, education level, rural residence. Clinical variables included: underlying chronic disease, preventive health practices, health status, health care worker, primary care provider, continuity of care, low birth weight, respiratory illness

**Table 3:** Association between influenza vaccination and SES, by three-measure SES

Study; Country	SES Concept (Level of SES measure)	Description SES measure	Adjusted mediating factors^a^	Data source; Data type (Year collected)	Results
Education, Income, and Occupational Class measured separately (*n* = 6; 5 mixed findings, 1 negative association)					
Der-Martirosian 2013 [43]; US	Education and employment (Individual)	Level of education (<HS, HS, some college, college graduate) Income ($15,000–$35,000; $35,000 or above) Employment status (employed, unemployed)	Sociodemographic variables, source of care, and veteran status	National Health Interview Survey; National survey (2010)	Mixed findings. Adults had greater odds of H1N1 vaccination where they had some college (1.29OR, 1.07–1.55CI) or graduated college (1.80OR, 1.49–2.19CI), compared to persons with less than high school; only college graduation was associated with seasonal influenza vaccination (1.58OR, 1.34–1.86CI). Higher income was positively associated (1.24OR, 1.11–1.40CI) only with seasonal influenza vaccination. Employment status was not associated with either H1N1 or seasonal influenza vaccination.
Laenen 2015 [44]; Belgium	Education, employment, and income (Individual and household)	Education: lower (no HS diploma), HS diploma, higher (beyond HS) Family income: less than 1500 euros, 1500–3000 euros, more than 3000 euros Work situation: fulltime, part-time, no job	None reported	Survey, clinical, and administrative data; Questionnaire, medical charts, and registry data from University Hospitals in Leuven (2013–14)	Mixed findings. Income or work situation did not influence vaccine coverage. Higher education pregnant women were more likely (2.73OR, 1.46–5.29CI) to be vaccinated than those with secondary school.
Liu 2012 [45]; Canada	Neighbourhood level of education, income, and employment (Neighbourhood)	Level 1 (lowest) to 5 (highest) assigned to neighbourhoods based on linked postal code records with Census data for highest level of education, median family income, and employment income	Sociodemographic variables, health status variables, clinical variables related to pregnancy, and neighbourhood-level characteristics	Better Outcomes Registry & Network; Birth records database (2009–10)	Mixed findings. Women who gave birth in an Ontario hospital with the highest level of education and the highest level of income were more likely to receive influenza vaccination compared to women from lower levels. Employment level was not significantly associated with vaccination.
Shin 2012 [46]; Korea	Education, employment, income (Individual)	Education (HS and above, less than high school) Employment (employed or unemployed) Monthly income (equal to or more than 2 million KRW, less than 2 million KRW)	Sociodemographic variables, health status variables, cancer variables, and vaccine knowledge and beliefs variables	Korean National Cancer Centre Survey; Nationwide survey (July – October 2010)	Mixed findings. Among cancer patients age 18 years and older, higher vaccination rates were associated with higher levels of education (1.72 OR, 1.02–2.93 CI).
Shono 2015 [47]; Japan	Education, employment, income (Individual)	Schooling years of the respondent Annual household income quintile Mother’s employment (Unemployed, employed)	Sociodemographic variables and vaccination recommendation	Survey of Japanese parents with at least one child under 13 years of age; Survey conducted for study purposes (2013)	Mixed findings. After controlling for sociodemographic variables and vaccination recommendation from a physician, the only significant relationship found was between the second-highest income quintile compared to the lowest (0.64 beta coefficient, 0.07 to 1.20 CI).
Yang 2014 [48]; Korea	Education, income, and occupational class (Individual)	Education (less than HS, HS or more) Monthly income (2 million KRW or more, less than 2 million KRW) Occupational class (professional, service/manual worker, others)	Sociodemographic variables	Korean Community Health Survey; Nationwide survey (2008–2012)	Negative association. Across all seasons, lower levels of income, working in service or physical occupations, and lower levels of education were significantly associated with higher levels of vaccination among Korean adults.
Socioeconomic status, as a composite measure (*n* = 3; 2 positive association, 1 no association)					
Bohmer 2012 [49]; Germany	Socioeconomic status (Individual)	High, medium, or low (determined based on education, income, and professional education)	Sociodemographic and health status variables	Germany Health Update; National survey (2009–10)	Positive association. High SES adults were more likely (1.61OR, 1.23–2.11CI) to be vaccinated against pandemic influenza compared to low SES adults.
Maher 2013 [50]; Australia	Socio-economic index summarizing information about the economic and social conditions of people and households within an area (Neighbourhood)	Index of Relative Socio-economic Disadvantage	Sociodemograhpic and antenatal care experience variables	Survey of women who delivered a baby in public hospitals; South Western Sydney and Sydney local health districts (2012)	No association. There was no significant association between level of SES disadvantage and influenza vaccination after adjusting for sociodemographic and antenatal care variables.
Schwartz 2013 [51]; Israel	Socioeconomic status (Neighbourhood)	Defined by the income quartile assigned by the zip code of the patient’s residence, using census data (High SES = 4, low SES = 1)	Sociodemographic variables, primary care variables, and comorbidities	Data from Maccabi Health Services; Maccabi Health Services (2004–09)	Positive association. There was a positive stepwise relationship between SES status and influenza vaccination status, whereby persons in high SES at Level 4 SES (ref) were more likely to be vaccinated compared to Levels 3 (0.82 OR, 0.79–0.85 CI), 2 (0.74OR, 0.71–0.77 CI), or 1 (0.72 OR, 0.68–0.77 CI) among the elderly aged 65 years and older.
Deprivation index (*n* = 3; 3 positive association)					
Brien 2012 [52]; Canada	Material and social deprivation (Neighbourhood)	Deprivation quintile (1 = low to 5 = high) derived from Pamplaon and Raymond’s index of material and social deprivation	Sociodemographic and clinical variables	Immunization records from National Public Health Institute of Quebec; Administrative data (2009)	Positive association. There were lower levels of vaccination in neighbourhoods with higher levels of material deprivation (per unit increase, approximately 7, 15, and 17% decrease in odds). No association was found between social deprivation and neighbourhood-level vaccination rates.
Calder 2014 [53]; New Zealand	Socioeconomic deprivation (Neighbourhood)	Level of socioeconomic deprivation, measured by NZDep 2006 quintile (1 = low, 5 = high deprivation)	None reported	Data from the patient management system of primary health organizations; Administrative data (2012–13)	Positive association. Vaccination among children improved after introducing a school vaccination programme. Vaccination was lowest in the most deprived quintiles (Dep1 = 30.2%, Dep2 = 29.2%, Dep3 = 36.6%, Dep4 = 24.3%, Dep5 = 21.9%)
Green 2015 [54]; England	Multiple deprivation (Neighbourhood)	Overall score assigned to each census lower super output area level summarizing relative deprivation based on: income, employment, health, education, crime, service, access, and living environment. Higher score = higher deprivation.	Population-level characteristics (e.g., sociodemographic, rural/urban classification)	Clinical and program data; 2–3 yr. olds: data collected from GP practices through ImmForm 4–11 yr. olds: data collected at schools from each of 6 pilot sites (2013–14)	Positive association. Children ages 4–11 were significantly less likely to be vaccinated in the two areas of highest deprivation, with scores of 26.1 to 39.9 (− 5.55 SC, − 9.54 to − 1.56CI) or 39.9+ (− 7.9 SC, − 12.16 to − 3.64 CI).

*Acronyms*: *CI* confidence interval, *NZDep* New Zealand Index of Deprivation, *OR* odds ratio, *HS* highschool, *PR* prevalence ratio, *SES* socioeconomic status, *US* United States

^a^Sociodemographic variables included: age, sex, place of residence, education level, rural residence. Clinical variables included: underlying chronic disease, preventive health practices, health status, health care worker, primary care provider, continuity of care, low birth weight, respiratory illness. Health belief variables included: knowledge, attitudes, practice

### Risk of bias across studies

The methodological quality of included studies was independently assessed two authors (KL, MLR) using critical appraisal tools developed by the Joanna Briggs Institute (JBI) for prevalence studies [55], analytical cross-sectional studies [56], and cohort studies [57]. It was decided that manuscripts would not be excluded based on their methodological quality, though the authors considered a manuscript to be of adequate quality where it received a minimum score of six out of nine, five out of eight, or seven out of 9, 8, or 11 (depending on the checklist), based on the experiences reported by others [58].

### Synthesis of results

A narrative summary of studies was completed. Study heterogeneity precluded the completion of a meta-analysis.

## Results

A total of 914 records were identified from the above search strategy, which resulted in 686 unique abstracts after the removal of duplicates. Five-hundred and ninety-four abstracts were excluded in the abstract review stage, leaving 92 articles for full-text review. Of these, 42 were synthesized and included in this review (Fig. 1).

### Study characteristics

Of the 42 studies that assessed the relationship between influenza vaccination and SES, 40 (95.2%) were descriptive or exploratory and two (4.8%) were analytical. Thirty-three studies (78.6%) were cross-sectional, five (11.9%) were ecological, and four (9.5%) were cohort studies.

Included studies predominantly came from the US (50.0%, *n* = 21), followed by South Korea (9.5%, *n* = 4), Canada (7.1%, *n* = 3), Belgium (4.8%, *n* = 2), Italy (4.8%, *n* = 2), and others (2.4%, *n* = 1 each from Australia, Denmark, England, Germany, Ireland, Israel, Japan, New Zealand, Poland, and Spain). Articles focused on a range of age groups. The majority focused on adult populations, defined as ranging from 18 to 65 years (38.1%, *n* = 16), or the general population (31.0%, *n* = 13). Other categories included children under 18 years (19.0%, *n* = 8) and the elderly over 65 years (11.9%, *n* = 5) of age. Specific subsets of populations included healthcare workers, parents, pregnant or postpartum women, or patients with diabetes, obesity, or multiple sclerosis.

### Risk of bias across studies

For the 37 studies assessed using the JBI Critical Appraisal Checklist for Prevalence Studies, the data quality scores were nine (100%) for 13 studies, eight (88.9%) for 17 studies, 7 (77.8%) for 4 studies, 6 (66.7%) for 2 studies, and 3 (33.3%) for 1 study. All four studies assessed using the JBI Critical Appraisal Checklist for Analytical Cross-Sectional Studies scored eight (100%). The single study assessed using the JBI Critical Appraisal Checklist for Cohort Studies received a data quality score of 10 (90.9%) out of 11. Disagreements on methodological quality were resolved through discussion and joint assessment until consensus was reached. Detailed results from quality assessments are provided in Additional files 2, 3, and 4.

### Relationship between SES and influenza vaccination

Included studies reported positive associations, negative associations, no association, or mixed associations.

Just over half of studies (52.4%, *n* = 22) [13, 14, 18–24, 26, 28–30, 37, 39–41, 49, 51–54], reported a positive association between high SES (any individual or combination of education, income, or occupation) and increased influenza vaccination. Nine of the 22 studies (40.9%) that reported positive associations used one measure to capture SES (e.g., education attainment level or household income level) [13, 14, 18–24], eight (36.4%, *n* = 8) used two or more measures of SES [26, 28–30, 37, 39–41], and five (22.7%, *n* = 5) used a single, composite measure of SES that combined the effects of education, income, and occupational class for an area (e.g., neighbourhood deprivation level) [49, 51–54].

Only two studies (4.8%) reported a negative association between SES and vaccination, where lower levels of education, income, or class resulted in higher rates of vaccination [17, 42, 48]. One of these studies reported a negative association using education only [17], while the other measured SES using a single composite measure that combined education, income, and occupational class [48].

Eleven studies (26.2%) reported mixed results [27, 31, 32, 34, 35, 38, 42–47]. All of the studies in this category used more than one measure to capture SES and reported inconsistent results on the association between influenza and SES. Seven studies used two measures [27, 31, 32, 34, 35, 38, 42] and five studies used three measures or a composite measure combining three or more measures of SES [43–47].

Six studies (14.3%) reported no association between SES and influenza vaccination. Of the six [15, 16, 25, 33, 48, 50], two used a single measure for SES [15, 16], three used two measures [25, 33, 36], and one used a single composite measure of SES that combined the effects of multiple socioeconomic variables within a neighbourhood [50].

### Relationship between SES measure and influenza vaccination

We found that most studies (*n* = 30, 71.4%) followed the recommended practice of measuring SES by using more than one measure of SES [8]. Twelve studies used three or more variables to measure SES; 18 used two variables to measure SES, using either a combination of education and income (*n* = 12) [27–37, 40], education and poverty (*n* = 3) [38, 39, 41], education and employment (*n* = 2) [25, 26], or income and poverty (*n* = 1) [42]; and 12 used a single measure for SES using education (*n* = 6) [13–18], income or poverty (*n* = 5) [20–24], or occupational class (*n* = 1) [19]. Specific details about the associations reported for each measure are reported in the sections that follow.

### Single measures of SES

Twelve of the 42 studies included in the review (28.6%) measured the relationship between influenza vaccination and one measure for SES: education, occupational class, or income. Of the 6 studies that used education as a single measure, 3 found a positive association. All 5 studies measuring income or poverty reported positive associations. The single study on occupational class found a positive association (Table 1).

#### Education (14.3%, *n* = 6/42)

Barbadoro et al. (2013) conducted a cross-sectional survey of Italian healthcare workers, among whom they found that lower levels of education were significantly associated with lower levels of influenza vaccination when compared to healthcare workers with post-graduate degrees, after adjusting for sociodemographic and clinical variables [13]. Cohen et al. (2012) found in a repeated cross-sectional study of adults in Washington Heights, New York that adults with higher levels of education were more likely to be vaccinated, with no relationship found for children [14].

In Spain and the US, two studies focused on influenza vaccination and SES in patient populations. A cross-sectional survey of Spanish adults with diabetes [16] found no significant association between levels of vaccination among persons with primary and secondary education after adjusting for age, sex, and other health and clinical variables. An historical cohort study of pregnant women ages 16 and older in Northern California [15], found an association between education and vaccination, where women with a master’s degree or higher were more likely to have been vaccinated compared to women with high school or less; however, this association disappeared after researchers adjusted for sociodemographic and maternal characteristics, including race, age, and provider recommendation for vaccine. Lorenz et al. (2013) [17] found that, after adjusting for age, health perception, and behaviour variables, education was negatively associated with influenza vaccination in adult outpatients with mental illness in Alabama. Specifically, outpatients with more than a high school level of education were significantly less likely to be vaccinated when compared to those with less than a high school education (0.29 OR; 0.09–0.96 CI, *p* < 0.01) [17]. Simon et al. (2016) conducted a cross-sectional study among children aged two to 17 years with asthma in the US, using the parents’ highest level of education as a measure of SES [18]. They found that children of parents with less education were significantly less likely to receive a vaccination as compared to those whose parents had at least one college degree (0.62 OR; 0.42–0.91 CI, *p* < 0.05) [18].

#### Occupational Class (2.4%, *n* = 1/42)

One historical cohort study of pregnant women in Dublin, Ireland found that during the H1N1 pandemic, women working in higher occupational classes (i.e., professional, manager, employer) were significantly more likely to be vaccinated compared to women working in any other occupation or who were unemployed (e.g., home duties, non-manual, manual, unemployed, non-classable) [19].

#### Income or poverty (11.9%, *n* = 5/42)

Five studies assessed the relationship between income and influenza vaccination. One cohort study from Ontario, Canada, determined that children from neighbourhoods with higher incomes were significantly more likely to be fully vaccinated against influenza than children from other areas [20]. Consistent with the above finding, an ecological study in New York City also found that children from neighbourhoods with higher levels of poverty had higher levels of H1N1 vaccination [23].

Fox and Shaw (2014), in a cross-sectional survey of the US general population, found that persons with higher family incomes were significantly more likely to have received an influenza vaccine [21]. Lau et al. (2013) likewise found higher income to be associated with significantly higher levels of vaccine in a cross-sectional survey of young adults aged 18 to 26 in California, though the association lost significance for income levels greater than 300% the federal poverty line [22]. Finally, Villarroel et al. (2016) found a positive association among US adults with diabetes, where vaccination levels were highest among the wealthiest of the sample [24].

### Two measures of SES

Eighteen of the 42 studies (42.9%) measured the relationship between influenza vaccination and SES using two measures (i.e., education and social or occupational class; education and income; education and poverty or income; income and poverty). Table 2 illustrates findings for all studies that used two measures to capture SES.

#### Education and social or occupational class (4.8%, *n* = 2/42)

Two studies measured SES using a combination of education and social or occupational class. In the US, LaVela et al. (2012) found a positive relationship between being vaccinated and being employed or having more education in a national survey of men with multiple sclerosis [25]. Lu et al. (2015) also conducted a cross-sectional survey of the US adult general population and reported a consistent and significant positive association between higher education levels, employment, and vaccination status [26]. For adults over 65, unemployment was associated with higher vaccination rates [26].

#### Education and income or poverty (35.7%, *n* = 15/42)

A cross-sectional survey of Italian adults found that obese persons aged 18 to 64 were significantly less likely to be vaccinated if they had completed high school, compared to those with only primary or intermediate schooling (0.77 OR; 0.62–0.96 CI, *p* < 0.05) [27]. A similar association was found for obese persons aged 64 and older [27]. No association was found when SES was measured using wealth (i.e., as an indicator of social class) for either age group [27]. In the US, one cross-sectional study of the general population found that children were significantly less likely to have received their first pH1N1 vaccination in households where the highest level of education was less than a college degree or where the household income was below four times the federal poverty line [28]. No relationship was found for the second pH1N1 vaccination. Schuller et al. (2013) found that children aged 19 to 35 months whose mothers had a college education or lived above the poverty level were significantly more likely to be immunized compared to those whose mothers had less education or lower levels of income [39]. Similarly, Zhai et al. (2017) reported that the likelihood of vaccination increased incrementally for US children aged 6 months to 8 years for each level of maternal education [41]. This association was found for the 2012–2013 flu season, but not for 2013–2014 [41]. Children from households with incomes greater than $75,000 per year were significantly more likely to have been vaccinated in both flu seasons [41].

A cross-sectional study conducted by the US Centers for Disease Control and Prevention (2013) found that pregnant women in Massachusetts with higher levels of education or income had higher levels of seasonal influenza vaccination, but not pH1N1 vaccination [29]. In New York State, a cross-sectional survey of postpartum women 14 to 47 years found that those with a graduate degree and the highest incomes were most likely to have received an H1N1 vaccination, which was tied to a healthcare provider recommendation in as many as 69% of cases [30]. When the authors examined the combined effects of income and education, no significant association was found [30].

Two studies compared US healthcare workers’ coverage to the general population. Lu et al. (2012) found that both populations were significantly more likely to receive H1N1 or seasonal influenza vaccination where they had higher levels of income and education [37]. In a similar study, researchers found that for healthcare workers and non-healthcare workers, higher levels of vaccination were significantly associated with having less than a high school level of education [38]. In addition, non-healthcare workers living at or above the poverty line were more likely to be vaccinated than persons below the poverty line [38].

In a cross-sectional survey of an elderly population with Type 2 diabetes in Lodz, Poland, Gorska-Ciebiada et al. (2015) found no significant association between education level and receipt of the 2012–2013 influenza vaccination [31]. However, adults with higher incomes (> 2000 Polish Zloty) were over five times as likely to be vaccinated than those with lower incomes [31]. A cross-sectional survey of the elderly in Denmark found no significant relationship between seasonal influenza vaccination and education or income level [32]. The same was found among the Belgian elderly [33], even after adjusting for the effects of age, sex, sociodemographic variables, health status, and risk factors. In Korea, Kwon et al. (2016) reported a significant and positive association between vaccination levels and high levels of education among an elderly population (1.27 OR; 1.03–1.57 CI, *p* = 0.025) after adjusting for sociodemographic variables, health status, and behavioural risk factors, but no association between income and vaccination status [34].

Lee et al. (2015) conducted a cross-sectional survey among the general population in South Korea and stratified results by age group [35]. Among persons aged 50 and older, vaccination levels decreased as education levels and household income levels increased; this association was found to be insignificant upon adjusting for the effects of age, sociodemographic, residence, health, and other factors [35]. Among the younger population, education levels and vaccination were negatively associated, but not statistically significantly [35]. However in the same study, higher household income levels and vaccination were positively associated. Lee et al. (2012) surveyed Korean-American women living in California and found no association between either education or income and influenza vaccination [36].

Takayama et al. (2012) found that among the elderly in the US, higher levels of education were positively and significantly associated with vaccination, but not for adults aged 18 to 64 [40]. Both in the older and the younger age groups, participants were significantly more likely to be vaccinated where they had higher levels of income [40].

#### Income and poverty (2.4%, *n* = 1/42)

One ecological study compared H1N1 vaccination rates among Minnesota residents by using an area-based measure [42]. The authors (2013) found that areas with greater proportions (8% of more) of persons living below the poverty line had higher vaccination rates than areas with lower proportions (5–8%)of persons living in poverty [42]. In areas where vaccination rates were low (< 20%), vaccination was associated with lower levels of family income [42]. In areas where vaccination rates were higher (>/=20%), higher incomes were associated with vaccination [42].

#### Income and class (0%, *n* = 0/42)

No studies measured SES using income and class.

### Three measures of SES

#### Education, income, and class (28.6%, *n* = 12/42)

Twelve studies measured the relationship between vaccination and SES using measures for education, income, and class; half combined all three measures as a composite score to report SES as a single measure, and half analyzed the three measures separately. The findings from studies that use three measures to capture SES are synthesized in Table 3.

Three of the studies using composite scores were ecological studies, which all found that higher SES was significantly associated with higher levels of vaccination. One ecological study conducted in Montreal, Canada found that pandemic coverage decreased where there were greater levels of material and social deprivation [52]. After adjusting for sociodemographic and health variables, only material deprivation remained associated with vaccination coverage, particularly for pregnant women, chronically ill older persons, and healthcare workers [52]. Another ecological study evaluated the effects of a program for children under 18 in Canterbury, New Zealand [53]. They found that persons better off socioeconomically had higher levels of vaccination. Before the program, vaccination rates had been lower, but more equally distributed across deprivation quintiles. Another ecological study, conducted among English children 2 to 11 years, found that higher levels of deprivation resulted in lower levels of vaccination [54]. For children 4 to 11 years, this relationship was only significant for the two most deprived groups [54].

Three cross-sectional studies used composite measures of SES, two of which reported a significant and positive relationship with influenza vaccination. A survey of the German adult general population, found that higher socioeconomic status was significantly associated with seasonal influenza vaccination in the post-pandemic season [49]. In Israel, Schwartz et al. (2013), also found a positive and significant association between SES and influenza vaccine among the elderly [51]. Maher et al. (2013) found no association between vaccination and socioeconomic disadvantage among women who delivered a baby in public hospitals of Sydney, Australia after adjusting for sociodemographic, antenatal care, and experience factors [50].

Of the studies that employed three measures of SES to separately capture the effects of education, income, and employment or class, four reported mixed positive and negative associations and two reported consistent associations. Among US adults, Der-Martirosian et al. (2013) found that education level was positively associated with H1N1 and seasonal influenza vaccination, while income was only associated with seasonal influenza vaccination, and employment status was associated with neither [43]. In Leuven, Belgium, a survey of pregnant women in their third trimester found that only education was positively and significantly associated with vaccination [44]. A cohort study of pregnant women in Ontario, Canada found that higher neighbourhood levels of education, income, and employment were all associated with higher levels of influenza vaccination (though employment was not associated with statistical significance) [45]. A Japanese survey of parents with one or more children under 13 years found that only household income was significantly associated with seasonal influenza vaccination, even though the mother’s employment and years of education were also included in the analysis [47].

Two studies of Korean populations both consistently reported associations across the separate measures of SES used. Among adult cancer patients, Shin et al. (2012) reported no significant association for employment, education, or income with vaccination [46]. Yang et al. (2014) found that vaccination levels were higher among persons with lower incomes, in non-professional occupations, and having less than a high school education across four flu seasons among adults 19 years and older [48].

### Public insurance status

Seventeen studies considered whether public funding or insurance programs might influence the association between SES and influenza vaccination. In the US, individuals receive health coverage privately through an insurance plan, publicly through Medicaid (adults) or other programs, or are uninsured. One US study found a negative association, where pregnant Medicaid beneficiaries were less likely to receive an influenza vaccine [29]. Nine other US studies and one Irish study reported a positive association between having insurance and being vaccinated among children and youth [18, 22, 28, 39], adults [43], pregnant women [19, 30], healthcare personnel [37, 38], the elderly [26, 40], and mental health patients [17]. Overall, the literature seems to show that in countries without universal publicly-funded insurance, persons with private insurance were more likely to be vaccinated than persons receiving public insurance through social programs, and, those with public insurance were more likely to be vaccinated than those without any coverage. Five of the above nine US studies adjusted for the effects of other variables (including SES) in their analysis, four of which found insurance coverage to be independently associated with influenza vaccination. Three studies considered the role of influenza vaccination and SES in countries with tax-funded healthcare systems (i.e., Denmark [32], Belgium [33], and Spain [16]) and found no association.

## Discussion

Over half of the studies included in this review support what has been reported previously: there is a relationship between SES and influenza vaccination. However, the direction of the relationship (i.e., positive or negative association) is not always clear and seems to vary depending on how SES is measured (e.g., as a single measure or a combination of education, income, class). The high numbers of positive associations likely reflect that nine of 42 studies (21.4%) reported this relationship using a single measure to capture SES. It is possible that the association may not have been so prominent, had additional measures been used. To illustrate the above point, consider the following two studies, both which used the US National Health Interview Survey. In 2011, Fox and Shaw (2014) found that higher income was associated with higher levels of vaccination [21]. In 2010, Der-Martirosian et al. (2013) found an association between higher income and vaccination, but reported an association with only some levels of education, and no association with employment status [43]. It is possible that the 2011 study would have found similar findings, putting into question the potential influence of “SES,” had they expanded their analysis.

Researchers may measure and report on SES depending on how they conceptualize this concept. Experts have recognized the implications that different measures of SES can have for healthcare policy and planning [59]. It is therefore important that authors employ as many measures as possible for SES and consider how they interact, to convey as much information about the influence of SES as possible.

In some cases, we found that while studies initially reported an association between SES and influenza, this association disappeared after adjusting for variables related to SES (e.g., age, sex, or rural location). One example is the relationship reported between education and influenza among pregnant women in California, which disappeared after the authors adjusted for race and healthcare provider recommendation [15]. These findings illustrate that factors other than SES may also affect the results and are important to consider in analysis due to their independent relationship with SES. For instance, individuals without public or private insurance may only access healthcare when they can afford to pay for it out of pocket. Similarly, individuals with higher levels of education may have access to jobs that afford them health insurance and access to healthcare providers.

Not all studies reported the variables that were adjusted for in the analysis, making it difficult to assess the true impact of SES on influenza vaccination. Experts have previously noted that SES is often employed as an adjustment variable in analysis to measure for potential confounding or effect measure modification [8], however, the above findings suggest it may be equally important to consider factors that are linked to SES less directly than income, education, and employment [60]. Additional factors that reflect an individual’s social position, including working conditions, job security, and social support may mitigate access to quality education, adequate income levels, and meaningful work. Other factors such as race (and racism), ability (and ableism), gender (and gender-based discrimination), and social stigma may affect an individual’s access to social and material resources.

Another variable linked to SES and influenza vaccination is insurance status, particularly in countries without universal, publicly-funded health care. We found that an overall positive relationship existed between persons with private health insurance and influenza vaccination in countries without universal, publicly-funded healthcare, but no association in countries with universal, publicly-funded health care. Studies from this review showed that private insurance was independently associated with influenza vaccination and SES, which suggests that its effects should be adjusted for during analysis as a potential confounder. Insurance status may serve as a marker of SES, since having private insurance may speak to the higher levels of skills or education that may be required to obtain well-paying, unionized, or other jobs likely to sponsor insurance plans for employees. Persons with health insurance may also be more likely to possess the financial and non-financial resources (e.g., transportation, time off work for appointments, health literacy, experience and trust in system) required to access healthcare. Some of the studies in this review have indicated that having a source of usual care and a physician that recommends influenza vaccination strongly influences whether individuals will receive immunization [16, 22, 34]. It is therefore recommended that authors explicitly address whether their study population is covered by publicly-funded health care or insurance programs when publishing on the topic of influenza vaccination and SES.

Finally, the studies reviewed show variation among different populations. Healthcare personnel in the US were more likely to be vaccinated if they had higher levels of education [37, 38]; however, this may also reflect the presence of mandatory vaccination policies in some healthcare settings, such as hospitals. Another difference was the findings among the elderly, which often contradicted those for younger adults [26, 40]. Trends in high-risk populations also differed from those in the general population. For example, while no association was found between income and vaccination for cancer patients in Korea or between education and vaccination among diabetic patients in Spain [16, 46]; a negative association was found between education and vaccination among obese patients in Italy [27]. It is possible that high-risk patients may be less affected by SES in situations where they receive regular care by a healthcare provider, which may – for populations with compromised immune systems – include a recommendation for vaccination.

There are limitations associated with this review. Studies were limited to the English language published between 2012 to 2017, which may have resulted in the exclusion of potentially relevant articles. A second limitation of this study is its focus on education, income, and class as determinants of SES. Other potential markers for SES include culture, race and ethnicity, knowledge, attitudes, and perceptions of the influenza vaccine, disability, and access to health care. Future research could focus on a broader assessment of SES to determine how the above factors mitigate the association between influenza vaccination and education, income, or class.

## Conclusion

This comprehensive review has contributed additional knowledge about the relationship between SES and influenza vaccination. To the best of our knowledge, this is the first systematic review to explore this topic without limiting studies to a specific population by age or characteristic. We found that a relationship appears to exist across different patient and sociodemographic populations, internationally, and that this relationship varies according to which measures of SES are used. Further research is needed to consider how factors related to SES beyond education, income, and class influence the relationship between SES and influenza. Finally, we recommend that authors be explicit in describing the SES concept they are trying to capture with the measure(s) they use when they assess the association between SES and influenza vaccination. Where possible, we recommend that multiple measures of SES be utilized to tell as complete a story as possible.

## Additional files

Additional file 1: Search strategies employed. This file contains the search strategies used for each database searched in the study. (DOCX 16 kb)

Additional file 2: Quality Assessment Results for Prevalence Studies (JBI Critical Appraisal Checklist for Studies Reporting Prevalence Data). A table reporting the results for each study assessed using the JBI Critical Appraisal Checklist for Studies Reporting Prevalence Data. (DOCX 35 kb)

Additional file 3: Quality Assessment Results for Analytical Cross-Sectional Studies (JBI Critical Appraisal Checklist for Analytical Cross-Sectional Studies) A table reporting the results for each study assessed using the JBI Critical Appraisal Checklist for Analytical Cross-Sectional Studies. (DOCX 18 kb)

Additional file 4: Quality Assessment Results for Cohort Studies (JBI Critical Appraisal Checklist for Cohort Studies) A table reporting the results for each study assessed using the JBI Critical Appraisal Checklist for Cohort Studies. (DOCX 20 kb)

## Data Availability

The full search strategy is available in Additional file 1. The quality assessments of included studies are available in Additional files 2, 3, and 4.

## Abbreviations

CI: Confidence Interval
JBI: Joanna Briggs Institute
OR: Odds Ratio
SES: Socio-Economic Status
US: United States of America
WHO: World Health Organization
