# Supplementary material for: The association between influenza vaccination and socioeconomic status in high income countries varies by the measure used: a systematic review
Source: BMC Med Res Methodol. 2019 Jul 17;19:153. doi: 10.1186/s12874-019-0801-1 (PMC6637551; doi:10.1186/s12874-019-0801-1)
Supplement: Supplementary file 1 — Search strategies employed. This file contains the search strategies used for each database searched in the study. (DOCX 16 kb) [file 12874_2019_801_MOESM1_ESM.docx]

**Additional File 1. Search strategies employed**

| **DATABASE** | **MEDLINE (OVID)** |
| --- | --- |
| **DATE** | **May 11, 2017** |
| #1 | (“social class” or “socioeconomic status” or “caste” or “occupational class” or “educational attainment” or “deprivation index” or “socioeconomic factors” or “inequity” or “inequality” or “employment” or “employment” or “social environment” or “social conditions” or “educational status” or “educational achievement” or “employment status” or “literacy” or “income” or “poverty” or “low income population” or “psychosocial deprivation” or “deprivation” or “vulnerable populations” or “sensitive population” or “disadvantaged” or “healthcare disparities” or “health status disparities” or “poverty areas” or “slum” or “ghetto”).ab,ti |
| #2 | (“social class” or “socioeconomic factors” or “social environment” or “social conditions” or “educational status” or “literacy” or “income” or “poverty” or “psychosocial deprivation” or “vulnerable populations” or “healthcare disparities” or “health status disparities”).sh. |
| #3 | 1 or 2 |
| #4 | influenza.ab,ti. |
| #5 | Influenza.sh. |
| #6 | 4 or 5 |
| #7 | (immuni* or vacci*).ab,ti. |
| #8 | (immunization or “immunization programs” or vaccination).sh |
| #9 | 7 or 8 |
| #10 | 3 and 6 and 9 |
| #11 | Limit 10 to English language |
| #12 | Limit 11 to 2012-2017 |
| #13 | limit 12 to (addresses or autobiography or bibliography or biography or comment or editorial or historical article or lectures or letter or news or newspaper article or patient education handout or personal narratives or portraits or video-audio media or webcasts) |
| #14 | 12 not 13 |
| #15 | Remove duplicates from 14 |

| **DATABASE** | **EMBASE (OVID)** |
| --- | --- |
| **DATE** | **May 11, 2017** |
| #1 | (“social class” or socioeconomics or “social status”).sh |
| #2 | ("health disparity" or "health care disparity" or "social isolation" or poverty or "educational status").sh |
| #3 | ("income" or "income group" or "highest income group" or "lowest income group" or "middle income group" or "family income" or "employment status" or "social environment" or "reading" or "vulnerable population").sh |
| #4 | 1 or 2 or 3 |
| #5 | (“social class” or socioeconomics or “social status”).ab,ti |
| #6 | ("income" or "income group" or "highest income group" or "lowest income group" or "middle income group" or "family income" or "employment status" or "social environment" or "reading" or "vulnerable population").ab,ti. |
| #7 | ("health disparity" or "health care disparity" or "social isolation" or poverty or "educational status").ab,ti. |
| #8 | 5 or 6 or 7 |
| #9 | 4 or 8 |
| #10 | influenza.ab,ti |
| #11 | Influenza.sh |
| #12 | 10 or 11 |
| #13 | (immuni* or vacci* or immunization or vaccination).ab,ti. |
| #14 | (immunization or "mass immunization" or vaccination or "influenza vaccination").sh |
| #15 | 13 or 14 |
| #16 | 9 and 12 and 15 |
| #17 | Limit 16 to English language |
| #18 | Limit 17 to 2012-2017 |
| #19 | limit 18 to (abstract report or books or "book review" or conference abstract or conference paper or "conference review" or editorial or erratum or letter or note or patent or tombstone) |
| #20 | 18 not 19 |
| #21 | Remove duplicates from 20 |
