# Supplementary material for: The association between influenza vaccination and socioeconomic status in high income countries varies by the measure used: a systematic review
Source: BMC Med Res Methodol. 2019 Jul 17;19:153. doi: 10.1186/s12874-019-0801-1 (PMC6637551; doi:10.1186/s12874-019-0801-1)
Supplement: Supplementary file 2 — Quality Assessment Results for Prevalence Studies (JBI Critical Appraisal Checklist for Studies Reporting Prevalence Data). A table reporting the results for each study assessed using the JBI Critical Appraisal Checklist for Studies Reporting Prevalence Data. (DOCX 35 kb) [file 12874_2019_801_MOESM2_ESM.docx]

**Additional File 2. Quality Assessment Results for Prevalence Studies (JBI Critical Appraisal Checklist for Studies Reporting Prevalence Data)**

| **Author Year** | **JBI Form used** | **1) Was the sample frame appropriate to address the target population?** | **2) Were study participants sampled in an appropriate way?** | **3) Was the sample size adequate?** | **4) Were the study subjects and the setting described in detail?** | **5) Was the data analysis conducted with sufficient coverage of the identified sample?** | **6) Were valid method used for the identification of the condition?** | **7) Was the condition measured in a standard, reliable way for all participants?** | **8) Was there appropriate statistical analysis?** | **9) Was the response rate adequate, and if not, was the low response rate managed appropriately?** | **Total checklist items (/9)** |
| --- | --- | --- | --- | --- | --- | --- | --- | --- | --- | --- | --- |
| Barbadoro 2013 (13) | **Prevalence** | **Y** | **Y** | **U** | **Y** | **Y** | **Y** | **Y** | **Y** | **Y** | **8** |
| Barbadoro 2016 (27) | **Prevalence** | **Y** | **Y** | **Y** | **Y** | **Y** | **Y** | **Y** | **Y** | **Y** | **9** |
| Blackwell 2015 (28) | **Prevalence** | **Y** | **Y** | **Y** | **Y** | **Y** | **Y** | **Y** | **Y** | **Y** | **9** |
| Bohmer 2012 (49) | **Prevalence** | **Y** | **Y** | **Y** | **Y** | **Y** | **Y** | **Y** | **Y** | **Y** | **9** |
| Brien 2012 (52) | **Prevalence** | **Y** | **Y** | **Y** | **Y** | **Y** | **Y** | **Y** | **Y** | **Y** | **9** |
| Calder 2014 (53) | **Prevalence** | **Y** | **Y** | **Y** | **Y** | **Y** | **Y** | **Y** | **Y** | **N/A** | **8** |
| Campitelli 2012 (20) | **Prevalence** | **Y** | **Y** | **Y** | **Y** | **Y** | **Y** | **Y** | **Y** | **Y** | **9** |
| CDC^*^ 2013 (29) | **Prevalence** | **Y** | **Y** | **Y** | **Y** | **Y** | **Y** | **Y** | **Y** | **Y** | **9** |
| Cleary 2014 (19) | **Prevalence** | **Y** | **Y** | **Y** | **Y** | **Y** | **U** | **Y** | **Y** | **Y** | **8** |
| Cohen 2012 (14) | **Prevalence** | **Y** | **Y** | **U** | **Y** | **Y** | **Y** | **Y** | **Y** | **Y** | **8** |
| Der-Martirosian 2013 (43) | **Prevalence** | **Y** | **Y** | **Y** | **Y** | **Y** | **Y** | **Y** | **Y** | **Y** | **9** |
| Dlugacz 2012 (30) | **Prevalence** | **Y** | **Y** | **U** | **Y** | **Y** | **Y** | **Y** | **Y** | **Y** | **8** |

| **Author Year** | | **JBI Form used** | **1) Was the sample frame appropriate to address the target population?** | **2) Were study participants sampled in an appropriate way?** | **3) Was the sample size adequate?** | **4) Were the study subjects and the setting described in detail?** | **5) Was the data analysis conducted with sufficient coverage of the identified sample?** | **6) Were valid method used for the identification of the condition?** | **7) Was the condition measured in a standard, reliable way for all participants?** | **8) Was there appropriate statistical analysis?** | **9) Was the response rate adequate, and if not, was the low response rate managed appropriately?** | **Total checklist items (/9)** |
| --- | --- | --- | --- | --- | --- | --- | --- | --- | --- | --- | --- | --- |
| Fox (2014) (21) | | **Prevalence** | **Y** | **Y** | **Y** | **Y** | **Y** | **Y** | **Y** | **Y** | **Y** | **9** |
| Gorska-Ciebiada 2015 (31) | | **Prevalence** | **N** | **U** | **U** | **U** | **U** | **Y** | **Y** | **Y** | **U** | **3** |
| Green 2015 (54) | | **Prevalence** | **Y** | **Y** | **U** | **Y** | **Y** | **Y** | **Y** | **Y** | **Y** | **8** |
| Jimenez-Trujilo 2015 (16) | | **Prevalence** | **Y** | **Y** | **Y** | **Y** | **Y** | **Y** | **Y** | **Y** | **Y** | **9** |
| Laenen 2015 (44) | | **Prevalence** | **U** | **Y** | **Y** | **Y** | **Y** | **Y** | **Y** | **Y** | **Y** | **8** |
| Lau 2013 (22) | | **Prevalence** | **Y** | **Y** | **Y** | **Y** | **Y** | **Y** | **Y** | **Y** | **U** | **8** |
| LaVela 2012 (25) | | **Prevalence** | **Y** | **U** | **U** | **Y** | **Y** | **Y** | **Y** | **Y** | **Y** | **7** |
| Lee 2012 (36) | **Prevalence** | | **Y** | **Y** | **U** | **Y** | **Y** | **Y** | **Y** | **Y** | **Y** | **8** |
| Liu 2012 (45) | **Prevalence** | | **Y** | **Y** | **Y** | **Y** | **Y** | **Y** | **U** | **Y** | **Y** | **8** |
| Lorenz 2013 (17) | **Prevalence** | | **U** | **Y** | **Y** | **Y** | **U** | **Y** | **Y** | **Y** | **U** | **6** |
| Lu 2012 (37) | **Prevalence** | | **Y** | **Y** | **Y** | **Y** | **Y** | **Y** | **Y** | **Y** | **U** | **8** |
| Lu 2015 (26) | **Prevalence** | | **Y** | **Y** | **Y** | **Y** | **Y** | **Y** | **Y** | **Y** | **N/A** | **8** |

| **Author Year** | **JBI Form used** | | **1) Was the sample frame appropriate to address the target population?** | **2) Were study participants sampled in an appropriate way?** | **3) Was the sample size adequate?** | **4) Were the study subjects and the setting described in detail?** | **5) Was the data analysis conducted with sufficient coverage of the identified sample?** | **6) Were valid method used for the identification of the condition?** | **7) Was the condition measured in a standard, reliable way for all participants?** | **8) Was there appropriate statistical analysis?** | **9) Was the response rate adequate, and if not, was the low response rate managed appropriately?** | **Total checklist items (/9)** |
| --- | --- | --- | --- | --- | --- | --- | --- | --- | --- | --- | --- | --- |
| Lu 2016 (38) | **Prevalence** | | **Y** | **Y** | **Y** | **Y** | **Y** | **Y** | **Y** | **Y** | **N/A** | **8** |
| Maher 2013 (50) | **Prevalence** | | **Y** | **Y** | **Y** | **Y** | **Y** | **Y** | **Y** | **Y** | **Y** | **9** |
| Muscoplat 2013 (42) | **Prevalence** | | **Y** | **Y** | **Y** | **Y** | **Y** | **Y** | **Y** | **Y** | **N/A** | **8** |
| Narciso 2012 (23) | **Prevalence** | | **Y** | **Y** | **N/A** | **Y** | **Y** | **Y** | **Y** | **Y** | **N/A** | **7** |
| Schuller 2013 (39) | **Prevalence** | | **Y** | **Y** | **Y** | **Y** | **Y** | **Y** | **Y** | **Y** | **N/A** | **8** |
| Schwartz 2013 (51) | **Prevalence** | | **U** | **Y** | **Y** | **Y** | **Y** | **Y** | **Y** | **Y** | **N/A** | **6** |
| Shin 2012 (46) | | **Prevalence** | **Y** | **Y** | **U** | **Y** | **U** | **Y** | **Y** | **Y** | **U** | **6** |
| Shono 2015 (47) | | **Prevalence** | **Y** | **Y** | **Y** | **U** | **Y** | **Y** | **Y** | **Y** | **U** | **7** |
| Simon 2016 (18) | | **Prevalence** | **Y** | **Y** | **Y** | **Y** | **Y** | **Y** | **Y** | **Y** | **U** | **8** |
| Takayama 2012 (40) | | **Prevalence** | **Y** | **Y** | **Y** | **Y** | **Y** | **Y** | **Y** | **Y** | **Y** | **9** |
| Villarroel 2016 (24) | | **Prevalence** | **Y** | **Y** | **Y** | **Y** | **Y** | **Y** | **Y** | **Y** | **Y** | **9** |
| Yang 2014 (48) | | **Prevalence** | **Y** | **Y** | **Y** | **Y** | **Y** | **Y** | **Y** | **Y** | **U** | **8** |
| Zhai 2017 (41) | | **Prevalence** | **Y** | **Y** | **Y** | **Y** | **Y** | **Y** | **Y** | **Y** | **Y** | **9** |

*CDC = Centers for Disease Control and Prevention
