# Supplementary material for: The association between influenza vaccination and socioeconomic status in high income countries varies by the measure used: a systematic review
Source: BMC Med Res Methodol. 2019 Jul 17;19:153. doi: 10.1186/s12874-019-0801-1 (PMC6637551; doi:10.1186/s12874-019-0801-1)
Supplement: Supplementary file 3 — Quality Assessment Results for Analytical Cross-Sectional Studies (JBI Critical Appraisal Checklist for Analytical Cross-Sectional Studies) A table reporting the results for each study assessed using the JBI Critical Appraisal Checklist for Analytical Cross-Sectional Studies. (DOCX 18 kb) [file 12874_2019_801_MOESM3_ESM.docx]

**Additional File 3. Quality Assessment Results for Analytical Cross-Sectional Studies (JBI Critical Appraisal Checklist for Analytical Cross-Sectional Studies)**

| **Author Year** | **JBI Form used** | **1) Were the criteria for inclusion in the sample clearly defined?** | **2) Were study subjects and the setting described in detail?** | **3) Was the exposure measured in a valid and reliable way?** | **4) Were objective, standard criteria used for measurement of the condition?** | **5) Were confounding factors identified?** | **6) Were strategies to deal with confounding factors stated?** | **7) Were the outcomes measured in a valid and reliable way?** | **8) Was appropriate statistical analysis used?** | **Total checklist items (/8)** |
| --- | --- | --- | --- | --- | --- | --- | --- | --- | --- | --- |
| Hellfritzsch 2017 (32) | **Cross-sectional (analytical)** | **Y** | **Y** | **Y** | **Y** | **Y** | **Y** | **Y** | **Y** | **8** |
| Hoeck 2013 (33) | **Cross-sectional (analytical)** | **Y** | **Y** | **Y** | **Y** | **Y** | **Y** | **Y** | **Y** | **8** |
| Kwon 2016 (34) | **Cross-sectional (analytical)** | **Y** | **Y** | **Y** | **Y** | **Y** | **Y** | **Y** | **Y** | **8** |
| Lee 2015 (35) | **Cross-sectional (analytical)** | **Y** | **Y** | **Y** | **Y** | **Y** | **Y** | **Y** | **Y** | **8** |
