# Supplementary material for: The association between influenza vaccination and socioeconomic status in high income countries varies by the measure used: a systematic review
Source: BMC Med Res Methodol. 2019 Jul 17;19:153. doi: 10.1186/s12874-019-0801-1 (PMC6637551; doi:10.1186/s12874-019-0801-1)
Supplement: Supplementary file 4 — Quality Assessment Results for Cohort Studies (JBI Critical Appraisal Checklist for Cohort Studies) A table reporting the results for each study assessed using the JBI Critical Appraisal Checklist for Cohort Studies. (DOCX 20 kb) [file 12874_2019_801_MOESM4_ESM.docx]

**Additional File 4. Quality Assessment Results for Cohort Studies (JBI Critical Appraisal Checklist for Cohort Studies)**

| **Author Year** | **JBI Form used** | **1) Were the two groups similar and recruited from the same population?** | **2) Were the exposure(s) measured similarly to assign people to both exposed and unexposed groups?** | **3) Was the exposure measured in a valid and reliable way?** | **4) Were confounding factors identified?** | **5) Were strategies to deal with confounding factors stated?** | **6) Were the groups / participants free of the outcome at the start of the study (or at the moment of exposure)?** | **7) Were the outcomes measured in a valid and reliable way?** | **8) Was the follow up time reported and sufficient to be long enough for outcomes to occur?** | **9) Was follow up complete, and if not, were the reasons to loss to follow up described and explored?** | **10) Were strategies to address incomplete follow up utilized?** | **11) Was appropriate statistical analysis used?** | **Total checklist items (/11)** |
| --- | --- | --- | --- | --- | --- | --- | --- | --- | --- | --- | --- | --- | --- |
| Henninger 2015 (15) | **Cohort** | **Y** | **Y** | **Y** | **Y** | **Y** | **Y** | **Y** | **U** | **Y** | **Y** | **Y** | **10** |
